# Fibulin‐4 as a potential extracellular vesicle marker of fibrosis in patients with cirrhosis

**DOI:** 10.1002/2211-5463.13842

**Published:** 2024-06-09

**Authors:** Masaru Kumagai, Atsunori Tsuchiya, Yuan Yang, Nobutaka Takeda, Kazuki Natsui, Yui Natusi, Kei Tomiyoshi, Fusako Yamazaki, Yohei Koseki, Hiroki Shinchi, Naoko Imawaka, Ryo Ukekawa, Takahiro Nishibu, Hiroyuki Abe, Takako Sasaki, Koji Ueda, Shuji Terai

**Affiliations:** ^1^ Division of Gastroenterology and Hepatology, Graduate School of Medical and Dental Sciences Niigata University Japan; ^2^ Future Medical Research Center for Exosome and Designer Cells (F‐DEC) Niigata University Japan; ^3^ Department of Gastroenterology, Digestive Disease Hospital Affiliated Hospital of Zunyi Medical University China; ^4^ Project for Realization of Personalized Cancer Medicine, Cancer Precision Medicine Center Japanese Foundation for Cancer Research Tokyo Japan; ^5^ Biotechnology Center, R&D Marketing Operations FUJIFILM Wako Pure Chemical Corporation Amagasaki‐shi Japan; ^6^ Department of Pharmacology, Faculty of Medicine Oita University Yufu Japan

**Keywords:** CD9, cirrhosis, extracellular vesicles, fibulin‐4, liver fibrosis

## Abstract

Chronic liver injury leads to decreased liver function and increased fibrosis. Fibrosis is not only associated with the development of portal hypertension and carcinogenesis, but with the occurrence of events and a poor prognosis, highlighting the importance of non‐invasive fibrosis assessment in patients. In the present study, we searched for markers related to liver fibrosis via proteomic analysis of small extracellular vesicles (sEVs). In the discovery cohort, proteomic analysis was carried out in the sEVs extracted from the sera of 5 patients with decompensated cirrhosis, 5 patients with compensated cirrhosis, and 5 controls without liver disease. Interestingly, in this cohort, fibulin‐4 was significantly associated with cirrhosis while in the validation cohort [formed by 191 patients: 7 patients without disease, 16 patients without liver disease (other diseases), 38 patients with chronic liver disease (CLD), 75 patients with cirrhosis of Child–Pugh class A (36 without hepatocellular carcinoma [HCC], 29 with HCC), and 65 patients with cirrhosis of Child–Pugh class B–C (39 without HCC, 26 with HCC)], fibulin‐4/CD9 levels increased with cirrhosis progression. Furthermore, the fibulin‐4/CD9 ratio was significantly higher in patients with varices. Immunostaining also revealed strong fibulin‐4 expression in cholangiocytes within the fibrous areas and mesothelial cells in liver tissue blood vessels. Taken together, our results suggest that fibulin‐4, essential for lysyl oxidase activation, might be a new liver fibrosis marker found in the sEVs of patients with cirrhosis.

## Graphical abstract

A potential fibrosis marker fibulin‐4 was discovered in extracellular vesicles from the serum of patients with liver cirrhosis. Fibulin‐4 expression tends to increase with fibrosis progression and is involved in lysyl oxidase activation. Fibulin‐4 is expressed in cholangiocytes and mesothelial cells within fibrotic areas in liver.

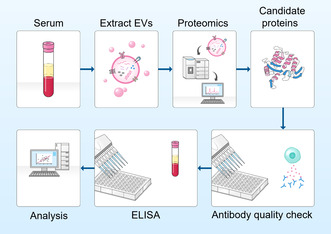

**Abbreviations**
ATX: autotaxin
AUC: Area Under the ROC Curve
CLD: chronic liver disease
ECM: extracellular matrix
EFEMP2: EGF‐containing fibulin extracellular matrix protein 2
ELISA: enzyme‐linked immunosorbent assay
FIB‐4: Fibrosis‐4 index
HCC: hepatocellular carcinoma
LOX: Lysyl oxidase
M2BPGi: macrophage galactose‐specific lectin‐2‐binding protein glycosylation isomer
P‐III‐P: Type III procollagen N‐terminal peptide
pro‐C3: N‐terminal type III collagen propeptide
ROC: receiver operating characteristic
sEVs: small extracellular vesicles

Chronic liver injury can lead to liver dysfunction and fibrosis. Progressive fibrosis leads to portal hypertension, which in turn alters circulatory dynamics, resulting in ascites, portosystemic shunts, and varices.^1^, ^2^ Furthermore, intestinal edema caused by portal hypertension can give rise to a variety of pathological conditions, including changes in the immune system and metabolic disorders. This triggers systemic inflammation and the invasion of bacteria through the intestinal barrier, eventually affecting multiple organs and resulting in a decreased quality of life and, in some cases, death.^3^

In the past, liver biopsy was the mainstay for diagnosing fibrosis. However, a wide variety of non‐invasive methods are currently used, ranging from blood tests, such as type IV collagen 7S,^4^ hyaluronic acid,^5^ type IIII procollagen N‐terminal peptide (P‐III‐P),^6^ N‐terminal type III collagen propeptide (pro‐C3),^7^ platelet count,^8^ autotaxin (ATX),^9^ and macrophage galactose‐specific lectin‐2‐binding protein glycosylation isomer (M2BPGi),^10^ to the FIB‐4 index,^11^ liver stiffness measurement using FibroScan^12^ and ultrasound,^13^ as well as magnetic resonance elastography (MRE).^14^, ^15^ While all fibrosis evaluation methods currently employed are excellent, there are variations and errors, highlighting the importance of combined diagnostic approaches.

Extracellular vesicles (EVs) are stable structures with a bilayer lipid membrane, approximately 100 nm in size, that are released from all cells to facilitate intercellular communication.^16^, ^17^, ^18^ They contain various nucleic acids and proteins, and their potential as biomarkers has been increasingly recognized. Small extracellular vesicles (sEVs), the main subset of EVs, are marked by CD9, CD63, and CD81. To date, no EV‐based fibrosis marker has been reported,^19^ as collecting EVs with high accuracy and evaluating them on a case‐by‐case basis is difficult.

Therefore, the current study aimed to reveal markers related to liver fibrosis through proteomic analysis of sEVs. We identified fibulin‐4 as a potential marker of liver fibrosis in sEVs.

## Materials and methods

### Proteomics samples, clinical data, and samples for discovery and validation cohort, and varices analysis

sEVs extracted from the sera of five patients with decompensated cirrhosis, five patients with compensated cirrhosis, and five controls without liver disease were used for proteomic analysis (Table 1). Subsequently, we used ELISA to analyze the frozen serum samples, tissue samples, and clinical data of 191 patients admitted to the Niigata University Medical and Dental Hospital, (Niigata, Japan) between April 2016 and March 2021. Seven patients without disease, 16 patients without liver disease (other diseases), 38 patients with chronic liver disease (CLD), 75 patients with cirrhosis of Child–Pugh class A [36 without hepatocellular carcinoma (HCC), 29 with HCC], and 65 patients with cirrhosis of Child–Pugh class B–C (39 without HCC, 26 with HCC) (Table 2). Fibrosis‐related markers were examined in some patients [platelets, fibrosis‐4 (FIB‐4) index, Mac‐2 binding protein glycosylation isomer (M2BPGi), and autotaxin (ATX) in 189, 189, 54, and 23 patients, respectively]. To analyze the association between varices and marker expression, all patients were divided into two groups: those with endoscopic esophageal, gastric, or duodenal varices (87 patients) and those without varices (104 patients).

**Table 1:** Baseline characteristics of the analyzed patients in discovery cohort. Alc, alcohol; IQR, interquartile range; MASH, metabolic dysfunction‐associated steatohepatitis; PBC, primary biliary cholangitis.

	Decompensated cirrhosis	Compensated cirrhosis	Control
Number of patients	5	5	5
Median (IQR) of age	57 (54–66)	66 (62–72)	29 (29–31)
Etiology (number)	MASH (2) MASH + Alc (1) Unknown (2)	PBC (1) MASH (1) Alc (2) Unknown (1)	ー
Child‐Pugh score (number)	Point 8 (2) Point 9 (1) Point 10 (1) Point 12 (1)	Point 5 (3) Point 6 (2)	ー

**Table 2:** Baseline characteristics of the analyzed patients in validation cohort. AIH, autoimmune hepatitis; ALB, albumin; Alc, alcohol; ALT, alanine aminotransferase; AST, aspartate aminotransferase; CLD, chronic liver disease; C‐P, Child–Pugh; FIB‐4, Fibrosis‐4 Index; HBV, hepatitis B virus; HCC, hepatocellular carcinoma; HCV, hepatitis C virus; IQR, interquartile range; LC, liver cirrhosis; M2BPGi, macrophage galactose‐specific lectin‐2‐binding protein glycosylation isomer; MASH, metabolic dysfunction‐associated steatohepatitis; PBC, primary biliary cholangitis; T‐Bil, total bilirubin.

	No disease	Other disease	CLD	LC C‐P A	LC C‐P B‐C
Number of patients	7	16	38	65	65
Median (IQR) of age	50 (44–67)	70 (61–78)	64 (49–70)	71 (63–77)	65 (55–73)
Etiology (number)	ー	ー	HBV (3) HCV (7) Alc (5) MASH (7) AIH (5) PBC (0) Others (11)	HBV (4) HCV (8) Alc (23) MASH (17) AIH (0) PBC (2) Others (11)	HBV (4) HCV (5) Alc (26) MASH (13) AIH (1) PBC (4) Others (12)
Number of Child‐Pugh grade, A/B/C	ー	ー	ー	65/0/0	0/44/21
Platlet count, median (IQR), x10^4^ per μL	29.1 (21.6–26.5)	21.2 (16.7–28.6)	20.4 (15.4–23.2)	9.9 (7.8–14.0)	8.5 (6.5–14.6)
AST, median (IQR), IU·L^−1^	25 (21–27)	24 (18–28)	26 (20–34)	35 (25–46)	50 (37–76)
ALT, median (IQR), IU·L^−1^	18 (17–22)	18 (14–28)	23 (16–40)	25 (16–37)	27 (22–45)
Alb, median (IQR), g·dL^−1^	4.6 (4.2–4.6)	4.0 (3.4–4.3)	4.2 (4.0–4.4)	3.9 (3.6–4.2)	2.8 (2.4–3.1)
T‐Bil, median (IQR), mg·dL^−1^	0.8 (0.5–0.9)	0.8 (0.5–0.9)	0.8 (0.7–0.9)	0.9 (0.7–1.4)	2.2 (1.3–4.0)
M2BPGi, median (IQR), C.O.I	0.45 (0.45–0.45)	0.37 (0.37–0.37)	1.08 (0.61–1.54)	3.60 (2.27–4.96)	9.99 (7.09–10.65)
FIB‐4 index, median (IQR)	0.83 (0.69–1.67)	1.29 (0.99–2.50)	1.52 (1.11–2.54)	4.48 (3.14–6.04)	7.35 (4.25–10.31)
Number of patients with HCC (−/+)	7/0	16/0	30/8	36/29	39/26
Number of patients with Varices (−/+)	7/0	16/0	37/1	30/35	14/51

This retrospective study was approved by the Institutional Review Board of Niigata University (2021–0032). Written informed consent was obtained from all patients. The study protocol conformed to the ethical guidelines of the 1975 Declaration of Helsinki.

### EV isolation and mass spectrometry analysis

EVs were isolated from 200 μL of serum samples using a MagCapture Exosome Isolation Kit PS (FUJIFILM Wako Pure Chemical Corporation, Osaka, Japan) according to the manufacturer's instructions, except that samples were eluted using 20 μL of 0.5% sodium dodecyl sulfate (SDS) in 50 mm triethylammonium bicarbonate.

After reduction with 10 mm TCEP at 100 °C for 10 min and alkylation with 25 mm iodoacetamide at ambient temperature for 45 min, protein samples were subjected to digestion with Trypsin/Lys‐C Mix (Promega Corporation, Madison, WI, USA) at 47 °C for 2 h on S‐Trap columns (ProtiFi, Fairport, NY, USA).

The resulting peptides were extracted from the gel fragments and analyzed using an Orbitrap Fusion Lumos mass spectrometer (Thermo Fisher Scientific, Waltham, MA, USA) combined with an UltiMate 3000 RSLC nano‐flow high‐performance liquid chromatographer (Thermo Fisher Scientific). Peptides were enriched using a μ‐Precolumn (0.3 mm i.d. × 5 mm, 5 μm, Thermo Fisher Scientific) and separated on an AURORA column (0.075 mm i.d. × 250 mm, 1.6 μm, Ion Opticks Pty Ltd, Fitzroy, Vic., Australia) using a two‐step gradient: 2–40% acetonitrile for 110 min, followed by 40–95% acetonitrile for 5 min in the presence of 0.1% formic acid. The compensation voltages for gas‐phase fractionation through a FAIMS Pro interface (Thermo Fisher Scientific) were set at −40, −60, and −80 V. The analytical parameters of the Orbitrap Fusion Lumos mass spectrometer were set as follows: resolution of full scans = 50 000, scan range (*m*/*z*) = 350–1500, maximum injection time of full scans = 50 ms, AGC target of full scans = 4 × 10^5^, dynamic exclusion duration = 30 s, cycle time of data‐dependent mass spectrometry (MS)/MS acquisition = 2 s, activation type = HCD, detector of MS/MS = Ion trap, maximum injection time of MS/MS = 35 ms, and AGC target of MS/MS = 1 × 10^4^.

The obtained MS/MS spectra were searched against the *Homo sapiens* protein sequence database in SwissProt using Proteome Discoverer 3.0 software (Thermo Fisher Scientific), in which peptide identification filters were set at “false discovery rate <1%.” Label‐free relative quantification analysis of proteins was performed using the default parameters of the Minora Feature Detector node, Feature Mapper node, and Precursor Ions Quantifier node in Proteome Discoverer 3.0 (Thermo Fisher Scientific).

### ELISA

EVs in patient sera were measured using the PS Capture™ Exosome ELISA Kit (FUJIFILM Wako Pure Chemical Corporation #298‐80 601) and biotinylated anti‐CD9 antibody (FUJIFILM Wako Pure Chemical Corporation #017‐28 211) as per the manufacturer's instructions, except for the use of biotinylated antibodies. An anti‐fibulin‐4 antibody (polyclonal mouse anti‐EFEMP2, purified immunoglobulin, Sigma‐Aldrich #SAB1400526‐50UG; We performed a fundamental primary investigation into the quantitative analysis of Fibulin‐4 using this antibody with EVs derived from cell lines expressing Fibulin‐4) was used. A Biotin Labeling Kit‐NH2 (#LK03, Dojindo Molecular Technologies, Inc., Kumamoto, Japan) was used to conjugate biotin to the anti‐fibulin‐4 antibody. This antibody was confirmed to extract sEVs from the culture supernatant of TIG3, a cell line expressing fibulin‐4, and to quantitatively measure sEVs. ELISA calculations were performed using an Infinite M Plex (Tecan Japan Co., Ltd., Kawasaki, Japan).

### Immunohistochemistry

For the staining of liver tissue, 10% formalin‐fixed tissue was cut into 4‐μm‐thick sections. Immunohistochemistry for fibulin‐4 (ab125073; Abcam, Cambridge, MA, USA; rabbit monoclonal antibody to fibulin‐4, dilution 1/250) was performed as follows: Dewaxed tissues were subjected to antigen retrieval in 10 mm sodium citrate buffer (pH 6.0) using a microwave for 20 min. Endogenous peroxidase activity was blocked via treatment with 3% hydrogen peroxide in phosphate‐buffered saline (PBS) for 10 min at room temperature. Anti‐fibulin‐4 antibody in PBS was added to sections for overnight incubation. Secondary antibody reactions were performed using the Vectastain ABC‐HRP kit (Vector Laboratories, Burlingame, CA, USA). The sections were then stained with DAB TRIS tablets (Muto Pure Chemicals, Tokyo, Japan).

### Statistical analyses

Data were processed, and volcano plots were created using graphpad prism v. 9.3.1 (GraphPad Software Inc., La Jolla, CA, USA). Data were assessed using the Mann–Whitney *U* test, and differences between groups were analyzed using one‐way analysis of variance. Differences were considered statistically significant at *P* < 0.05.

## Results

### Fibulin‐4 is strongly expressed in serum EVs from patients with decompensated cirrhosis in the discovery cohort

**Fig. 1:**
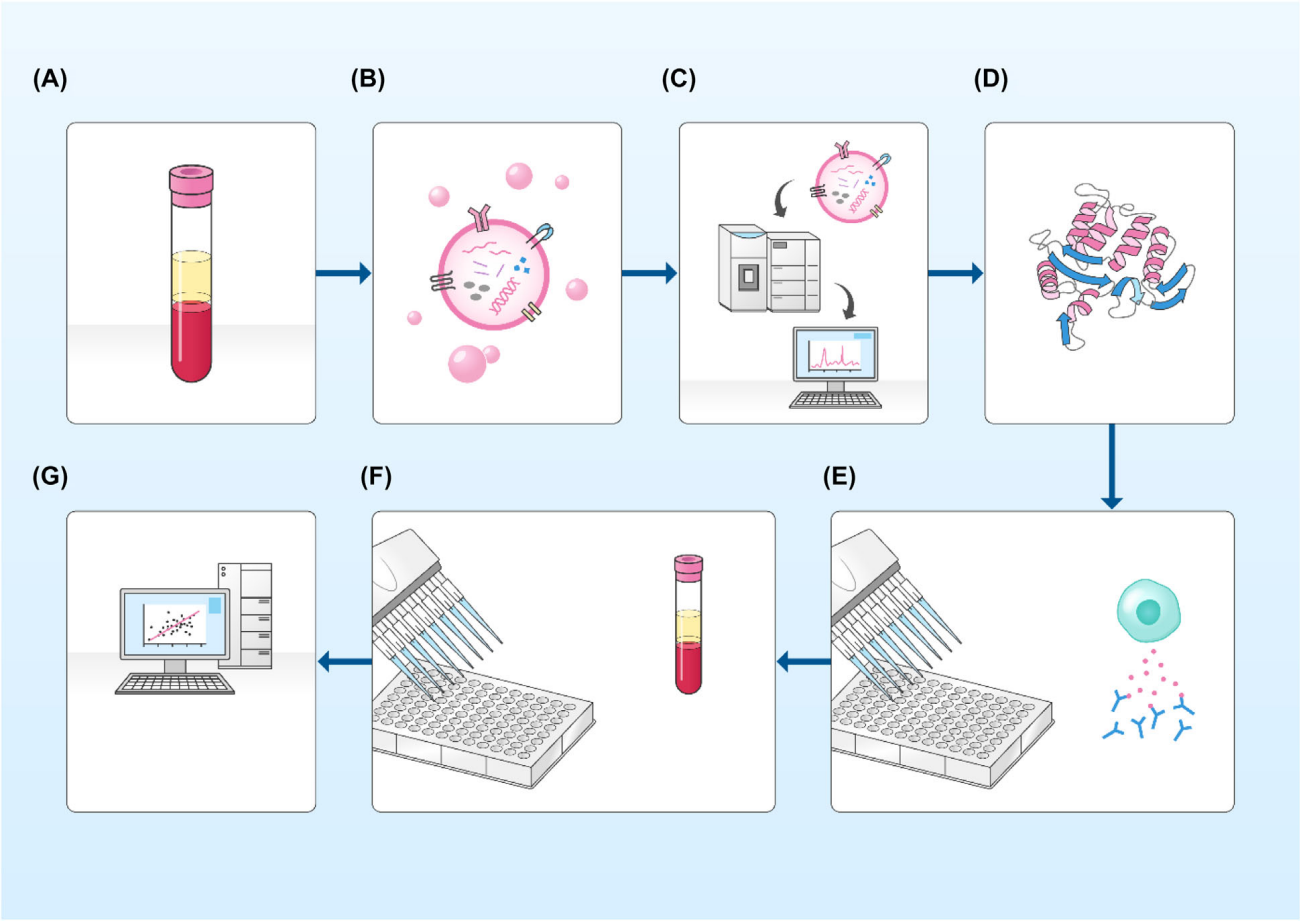
Flowchart of this experiment. (A) From serum, (B) we extracted extracellular vesicles, (C) performed proteomics analysis, and (D) selected candidate proteins. (E) We verified whether antibodies could be used to detect the target protein and then quantitatively measured target protein expression via enzyme‐linked immunoassay (ELISA). Finally, (F) a large number of samples were assayed using ELISA, and (G) associations with clinical data were determined.

We identified markers associated with fibrosis in the sEVs of patients with liver cirrhosis (Fig. 1A–G). First, we extracted sEVs from the sera of five patients with decompensated liver cirrhosis, five patients with compensated liver cirrhosis, and five controls without liver disease. These EVs were then subjected to proteomic analysis. We detected 2182, 2198, and 2068 EV‐derived proteins in the sera from patients with decompensated cirrhosis, compensated cirrhosis, and controls without liver disease, respectively.

**Fig. 2:**
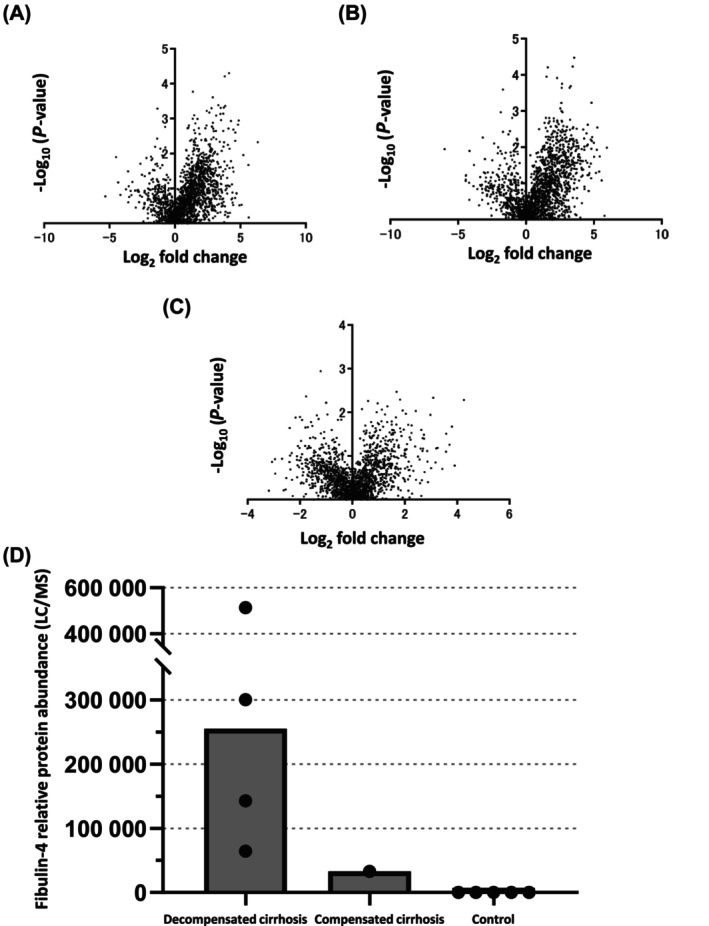
Proteomics analysis. Volcano plot showing protein expression in (A) control vs. patients with compensated cirrhosis, (B) control vs. patients with decompensated cirrhosis, and (C) patients with compensated cirrhosis vs. those with decompensated cirrhosis. (D) Representative results of fibulin‐4 expression as per proteomic analysis from five cases of each group.

We then compared protein expression between the groups, observing these via volcano plots (Fig. 2A–C). We noted significant changes in certain protein levels between patients with cirrhosis and controls. We focused on fibulin‐4 in particular, as it could not be detected at all in controls without liver disease. Meanwhile, fibulin‐4 was detected in four of the five patients with decompensated cirrhosis and one of five patients with compensated cirrhosis (Fig. 2D).

### Fibulin‐4/CD9 levels increase with cirrhosis progression in the validation cohort

**Fig. 3:**
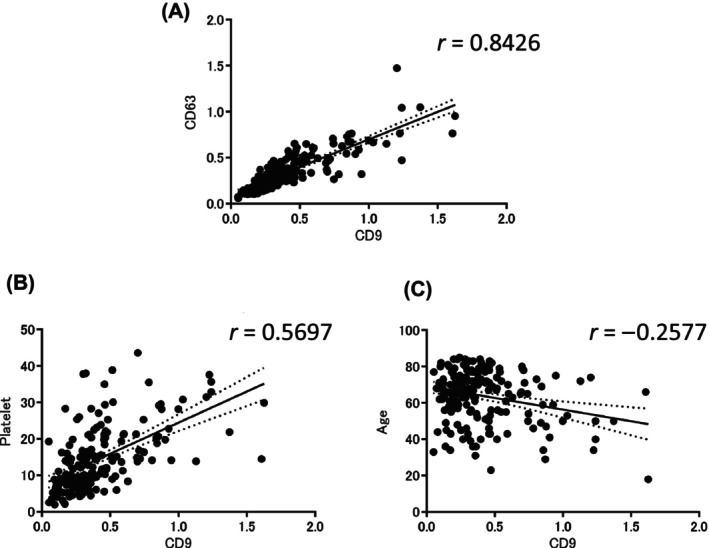
Association of extracellular vesicle CD9 vs. CD63 levels, platelets, and age. (A) Correlation of CD9 and CD63 levels in extracellular vesicles, (B) correlation between extracellular vesicle CD9 levels and platelets, and (C) correlation between extracellular vesicle CD9 levels and age.

Next, we performed ELISA using a biotinylated antibody confirmed to quantify EVs in a cell line expressing fibulin‐4. The kit we used could quantitatively measure common EV markers CD9 and CD63 in all serum samples analyzed in this study. We confirmed a strong positive correlation between CD9 and CD63 levels (*r* = 0.8426) (Fig. 3A). CD9 also showed a positive correlation (*r* = 0.5697) (Fig. 3B) with platelets and a weak negative correlation (*r* = −0.2577) (Fig. 3C) with age. This finding indicates that the amount of EVs may decrease with the progression of fibrosis and aging. Since the number of EVs differed across patients, we decided to normalize the fibulin‐4 level to that of CD9 per patient. We then measured serum samples from 191 patients: seven without disease, 16 without liver disease (other diseases), 38 with chronic liver disease (CLD), 65 with cirrhosis of Child–Pugh class A [36 without hepatocellular carcinoma (HCC), 29 with HCC], and 65 with cirrhosis of Child–Pugh class B–C (39 without HCC, 26 with HCC) (Table 2). The other (non‐liver) disease group included cases of acute pancreatitis, autoimmune pancreatitis, intraductal papillary mucinous neoplasms, interstitial pneumonia, focal nodular hyperplasia, enteritis, early gastric cancer, early esophageal cancer, infected pancreatic cysts, cholangitis, sarcoidosis, and lung infection.

**Fig. 4:**
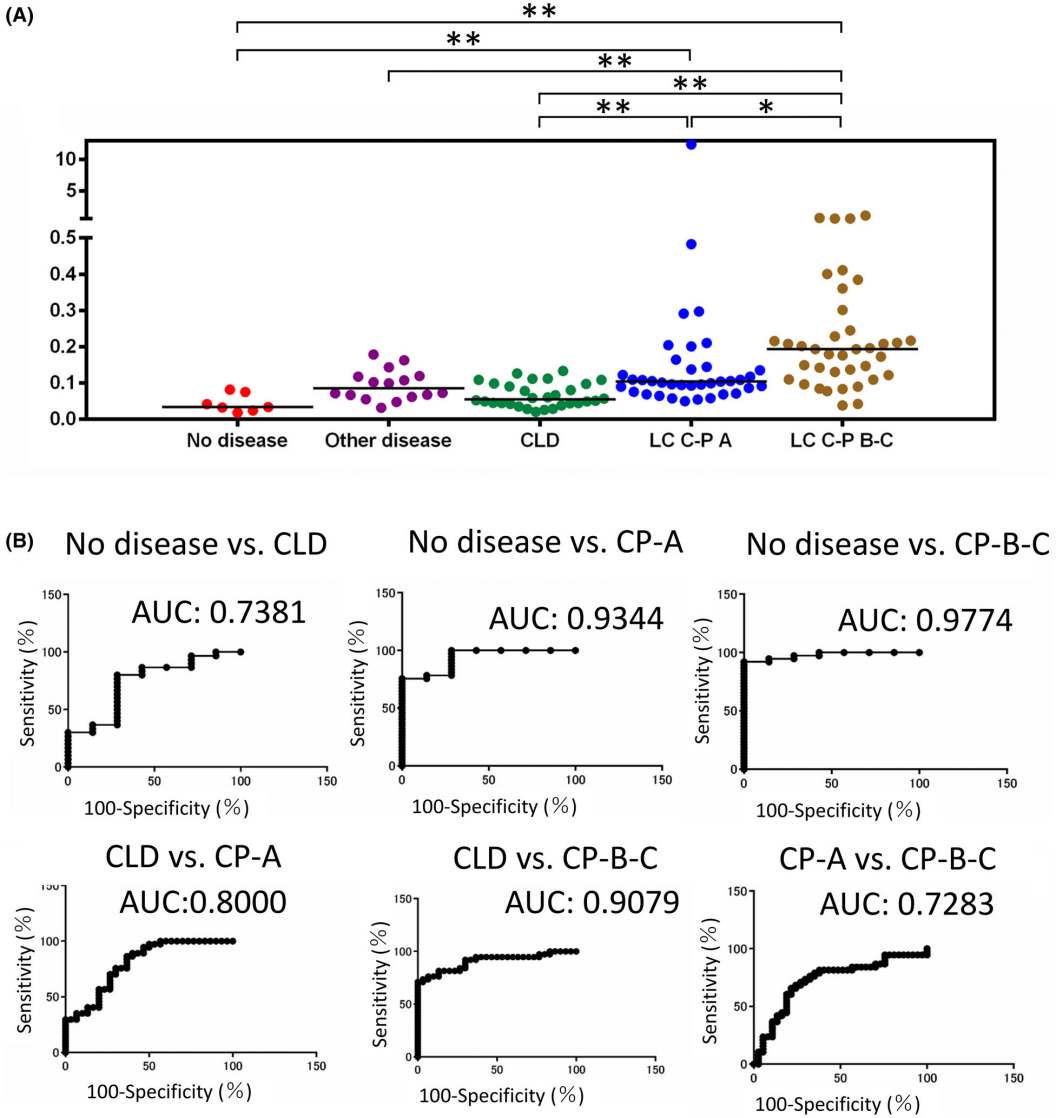
Fibulin‐4/CD9 ratio in serum extracellular vesicles based on disease state (excluding patients with hepatocellular carcinoma). (A) Fibulin‐4/CD9 ratio values for each disease state, excluding hepatocellular carcinoma. (B) Receiver operating characteristic (ROC) curves showing the comparison between disease states and the diagnostic accuracy of the fibulin‐4/CD9 ratio for distinguishing each disease state. *P < 0,05, **P < 0.01. AUC, area under the ROC curve; C‐P, Child–Pugh. [Correction added on 5 August 2024, after first online publication; Figure 4A has been updated to correct the alignment of the significance bars.]

First, we evaluated the association of the fibulin‐4/CD9 ratio with liver fibrosis in the absence of HCC in seven healthy patients, 16 patients without liver disease (other diseases), 38 patients with CLD, 36 patients with Child–Pugh class A cirrhosis (without HCC), and 39 patients with Child–Pugh class B–C cirrhosis (without HCC). The median fibulin‐4/CD9 ratio was found to increase as liver fibrosis progressed. A significant difference in the ratio value was found between CLD and Child–Pugh class A cirrhosis (*P* < 0.01), with an area under the receiver operating characteristic (ROC) curve (AUC) of 0.8000, as well as between CLD and Child–Pugh class B‐C cirrhosis (*P* < 0.01), with an AUC of 0.9079. A significant difference was also noted between the Child–Pugh class A and B‐C (*P* < 0.05) groups, with an AUC of 0.7283 (Fig. 4A,B).

**Fig. 5:**
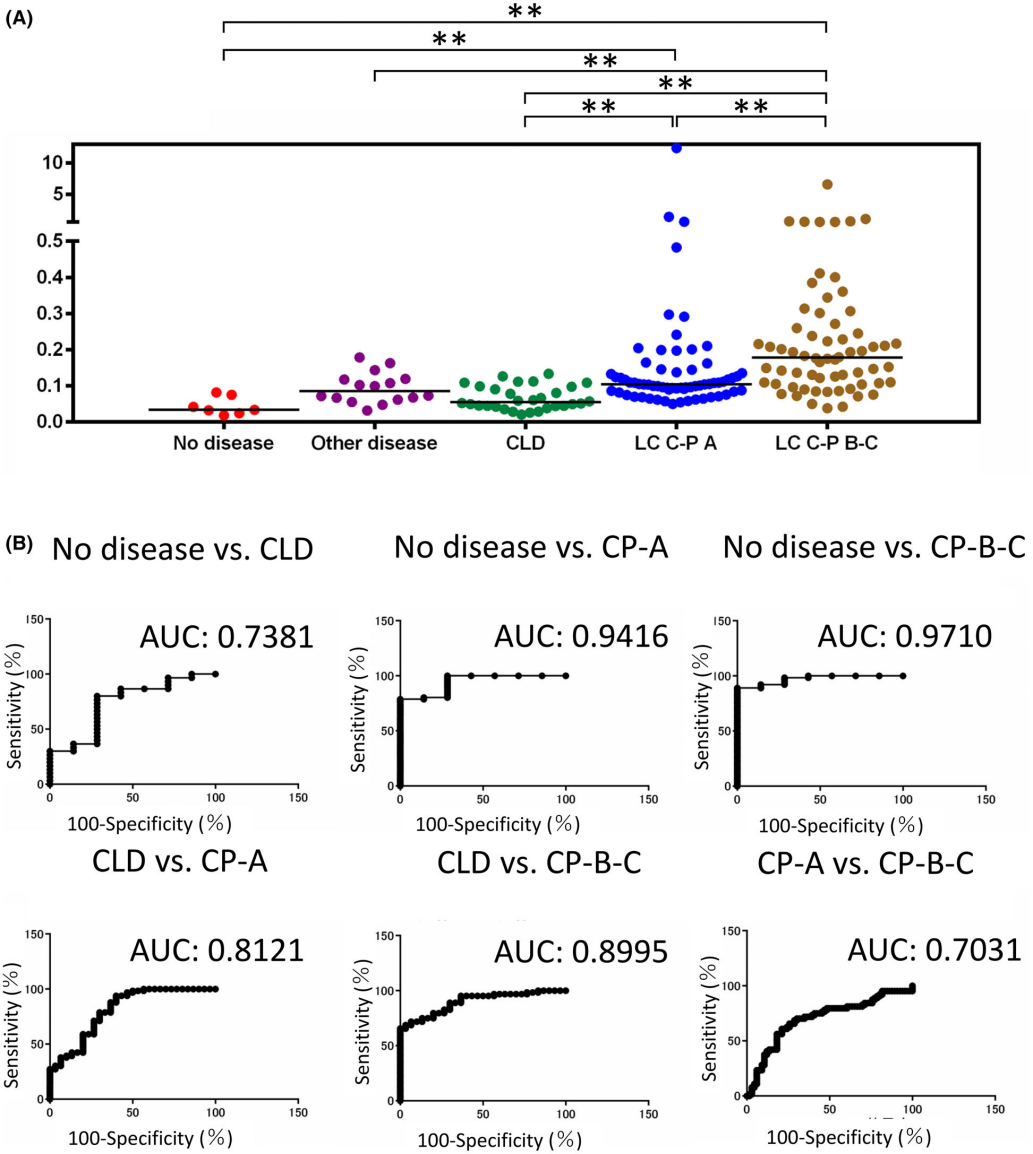
Fibulin‐4/CD9 ratio in serum extracellular vesicles by disease state (including patients with hepatocellular carcinoma). (A) Ratio values are measured for each disease state, including hepatocellular carcinoma. (B) Receiver operating characteristic (ROC) curves showing the comparison of each disease state and the diagnostic accuracy of the fibulin‐4/CD9 ratio for distinguishing between each disease state. **P < 0.01. AUC, Area under the ROC curve; C‐P, Child–Pugh. [Correction added on 5 August 2024, after first online publication; Figure 5A has been updated to correct the alignment of the significance bars.]

Next, all patients, including those with HCC, were analyzed. The median fibulin‐4/CD9 values increased as liver lesions progressed, with a significant difference between CLD and Child–Pugh class A (*P* < 0.01), with an AUC of 0.8121, as well as between CLD and Child–Pugh class B‐C cirrhosis (*P* < 0.01), with an AUC of 0.8995. Child–Pugh class A and Child–Pugh class B‐C (*P* < 0.01) also differed significantly, with an AUC of 0.7031 (Fig. 5A,B). Taken together, the fibulin‐4/CD9 ratio increased with the degree of fibrosis, irrespective of the presence or absence of HCC.

**Fig. 6:**
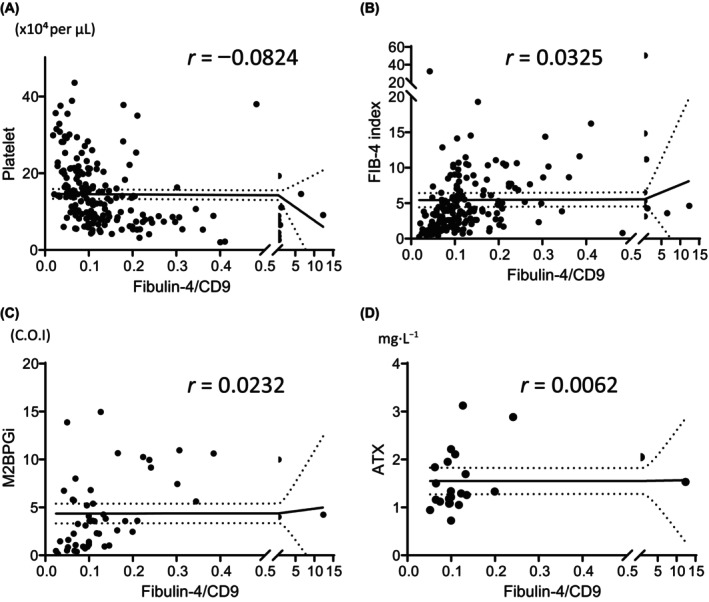
Association of the fibulin‐4/CD9 ratio with platelets, FIB‐4 index, M2BPGi, and ATX. (A) Correlation between the fibulin‐4/CD9 ratio and platelets (*n* = 189). (B) Correlation between the fibulin‐4/CD9 ratio and fibrosis‐4 (FIB‐4) index (*n* = 189). (C) Correlation between the fibulin‐4/CD9 ratio and Mac‐2 binding protein glycosylation isomer (M2BPGi) (*n* = 54). (D) Correlation between the fibulin‐4/CD9 ratio and Autotaxin (ATX) (*n* = 23).

Fibulin‐4/CD9 was compared with traditional indicators and the markers associated with fibrosis, including platelets (*r* = 0.0824), FIB‐4 (*r* = 0.0325), M2BPGi (*r* = 0.0232), and ATX (*r* = 0.0062). These findings indicate that a high correlation was not observed with any of the indices or markers (Fig. 6A–D).

### Varices are associated with a higher fibulin‐4/CD9 ratio

**Fig. 7:**
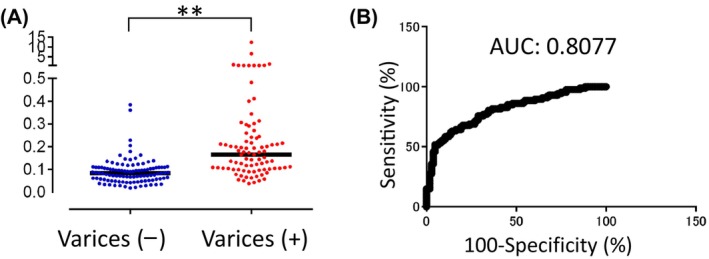
Differences in the fibulin‐4/CD9 ratio in serum extracellular vesicles from patients with and without varices. (A) Differences in the fibulin‐4/CD9 ratio between patients with and without varices. (Bars indicate the median.) (B) Receiver operating characteristic (ROC) curves representing the accuracy of the fibulin‐4/CD9 ratio for diagnosing varices [Varices (−); *n* = 104, varices (+); *n* = 87], **P < 0.01. AUC, Area under the ROC curve.

Next, all patients were divided into two groups: those with endoscopic esophageal, gastric, or duodenal varices (87 patients) and those without varices (104 patients). Patients with varices had a significantly higher fibulin‐4/CD9 ratio than those without (*P* < 0.001). A ROC curve was also generated, and the AUC of 0.8077 indicated that the ratio could discriminate well between patients with and without varices (Fig. 7A,B).

### Fibulin‐4 is upregulated in cholangiocytes within fibrotic areas and mesothelial cells in blood vessels

**Fig. 8:**
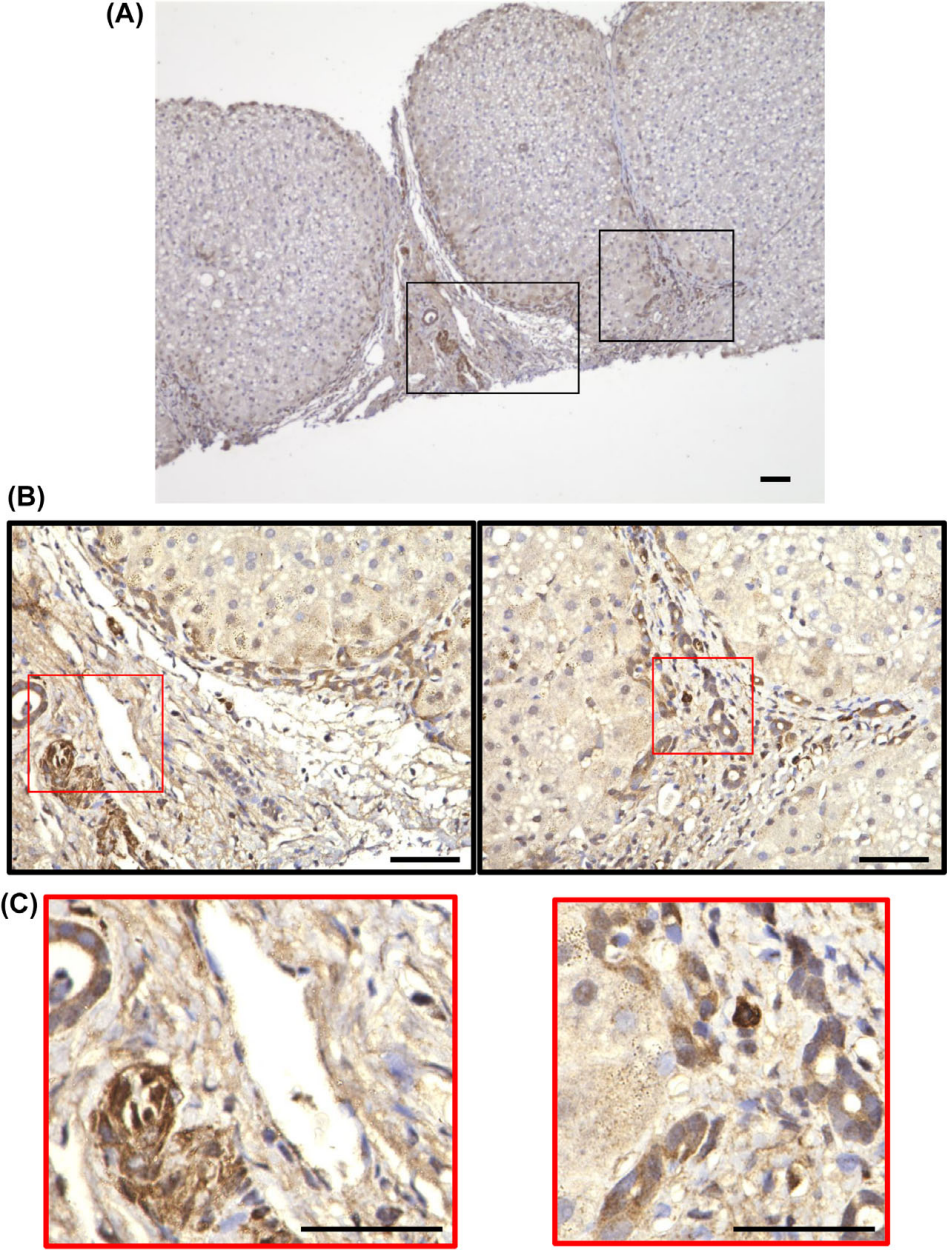
Immunohistochemical staining of fibulin‐4 in cirrhotic liver tissues. (A) Immunohistochemical staining of cirrhotic tissue with an anti‐fibulin‐4 antibody. (B) A magnified view of the black frame in (A). (C) Magnified view of the red frame in (B) Scale bar = 50 μm.

According to single‐cell analysis of the Protein Atlas,^20^ fibulin‐4 is expressed in fibroblasts, smooth muscle cells, endothelial cells, and cholangiocytes. To verify whether fibulin‐4 is indeed expressed in the liver and to determine which cell types express it, we performed staining of tissues from patients with liver cirrhosis. Fibulin‐4 expression was noted in bile duct cells near fibrotic regions in the liver and in the mesothelial cells of blood vessels (Fig. 8A–C).

## Discussion

In this study, we identified fibulin‐4 [also known as epidermal growth factor‐containing fibulin extracellular matrix protein 2 (EFEMP2)] as a novel fibrosis marker in serum sEVs. It was particularly upregulated in patients with decompensated liver cirrhosis in both the discovery and validation cohorts. In addition to its elevation with the progression of cirrhosis, the levels of this marker were significantly higher in patients with endoscopic varices than in those without varices, indicating portal hypertension. Within the liver, fibulin‐4 expression was observed close to regions of fibrosis, namely, the bile ducts and mesothelial cells of the arteries.

The matricellular protein fibulin‐4 is essential for lysyl oxidase (LOX) activation.^21^ This LOX family member cross‐links structural extracellular matrix (ECM) components, particularly fibrous collagen and elastin, being strongly implicated in fibrosis progression and resistance to fibrosis reversal. LOX proteins (LOX, LOXL1, LOXL2, LOXL3, and LOXL4) are extracellular copper‐dependent enzymes and have been identified as potential therapeutic targets in liver fibrosis. Further, fibulin‐4 has been reported to play a specific role in promoting the proper maturation of elastic fibers by linking LOX to tropoelastin through interactions with the LOX propeptide, which catalyzes the cross‐linking of elastin molecules.^22^ Fibulin‐4, a LOX‐related protein, differs from conventional indicators of fibrosis and does not correlate well with conventional indicators. Moreover, its significance may deepen in many clinical specimens in the future.

Fibulin‐4 is a member of the fibulin protein family composed of long (fibulin‐1, ‐2, ‐6) and short fibulins (fibulin‐3, ‐4, ‐5, ‐7) involved in protein–protein interactions between components of the basement membrane and ECM. Fibulin‐1, ‐2, ‐3, ‐4, and ‐5 bind the monomeric form of elastin (tropoelastin) *in vitro*. Further, fibulin‐2, ‐3, ‐4, and ‐5 are involved in various aspects of elastic fiber development *in vivo*. In particular, fibulin‐4 and ‐5 are non‐dispensable for elastic fiber assembly.^23^ Fibulin‐3, −4, and ‐5 are closely related paralogs with very similar domain structures and sequences, but have independent molecular functions in elastogenesis, as indicated by the distinct phenotypes noted in knockout mice and patients with mutations in the corresponding genes.^23^

More recently, evidence suggesting that fibulin‐4 is necessary for fibrillar collagen assembly has emerged.^23^ Fibulin‐4^−/−^ mice die immediately before birth because of arterial hemorrhage, while fibulin‐4^+/−^ mice appear normal.^24^ Insufficient levels of fibulin‐4 compromise the structural integrity of the aortic wall and can lead to aneurysm. Consistent with this, patients with mutations in fibulin‐4 suffer from cardiovascular complications, including aortic aneurysms, arterial tortuosity, and elastin abnormalities as described in detail previously.^25^ Mutations in fibulin‐4 also cause autosomal recessive cutis laxa 1B, characterized by loose skin, with vascular, lung, and skeletal abnormalities.

Although reports on the association of fibulin‐4 with liver disease are rare, Pantano *et al*. have identified gene expression clusters that strongly correlate with the fibrosis stage, including four genes consistently reported across previously published transcriptomic studies on metabolic dysfunction‐associated steatohepatitis: *COL1A2*, *EFEMP2* (fibulin‐4), *FBLN5* (fibulin‐5), and *THBS2*.^26^ This report supports our findings, suggesting that fibulin‐4 and fibulin‐5 are associated with liver fibrosis.

In this study, we detected fibulin‐4 in sEVs. In general, EVs disappear from the blood in a relatively short period and thus dynamically reflect fibrosis at any given time. However, we did not identify fibulin‐5 in sEVs. This suggests that members of the fibulin family may have different roles and kinetics, particularly in sEVs from patients with fibrosis.

Zhang *et al*.^27^ reported that fibulin‐4 promotes osteosarcoma invasion and metastasis by inducing the epithelial‐to‐mesenchymal transition (EMT) via the PI3K/Akt/mTOR pathway. Li *et al*.^28^ reported that EFEMP2 increases the invasive ability of cervical cancer cells by promoting EMT via the Raf/MEK/ERK signaling pathway. In contrast, Kang *et al*.^29^ reported that EFEMP2 inhibited breast cancer invasion and metastasis *in vitro* and *in vivo*.

Currently, various indices are available to evaluate fibrosis. Liver stiffness measurements using FibroScan, ultrasound, and MRE are widely used in clinical practice. One drawback of these existing methods is that they reflect inflammation. However, fibulin‐4 is theoretically related to fiber maturation and may be less susceptible to the effects of inflammation. In addition, unlike fibulin‐4, other markers are not related to fiber maturation. Thus, we believe that fibulin‐4 could be a marker that reflects the progression of liver stiffness in a clinical setting.

In this study, fibulin‐4 was expressed in cholangiocytes, particularly those undergoing ductular reactions, and in mesothelial cells of the surrounding blood vessels. The ductal cells undergoing ductular reactions are constantly present within fibrosis lesions. Fibulin‐4 may contribute to the stabilization of growing cholangiocytes and may initially play an important role in the tissue repair process, with excessive growth potentially leading to fibrosis.

The study had some limitations. It did not assess the samples for acute liver and non‐liver diseases. The clinical significance of this marker should be further explored. In future studies, samples from patients with acute liver and non‐liver diseases should also be assessed in order to clarify the expression patterns of this marker. Moreover, the diagnostic value of fibulin‐4 compared to other markers should be determined in patients with severe cirrhosis. The serum was not assayed in the present study owing to the unavailability of ELISA or other suitable assay systems. However, it may be more meaningful to evaluate whether the serum can be used to determine the difference in the future. The present study is novel in that it is the first study to use EVs to evaluate individual cases, which is technically difficult. The novelty of the present study lies in the fact that the ELISA method used herein was able to achieve this.

Despite these limitations, our identification of a new serum EV‐based marker of liver fibrosis is significant. We believe that fibulin‐4 has potential as a new biomarker of fibrosis in the clinic.

## Conflict of interest

Shuji Terai received the research funding from FUJIFILM Wako Pure Chemical Corporation. Atsunori Tsuchiya received research funding from Shionogi & Co., Ltd.

### Peer review

The peer review history for this article is available at https://www.webofscience.com/api/gateway/wos/peer-review/10.1002/2211-5463.13842.

## Author contributions

MK and AT collected and analyzed the data and wrote the manuscript. YY, NT, KN, YN, KT, FY, YK, YY, and TS collected and analyzed the data. NI, RU, and TN developed the ELISA and KU. performed the proteomics analysis. ST supervised the study. All the authors reviewed the manuscript.

## Data Availability

All data needed to evaluate the conclusions of this study are provided in the main text of the manuscript and/or in the Supplementary Materials. This study included no data deposited in external repositories. Additional data related to this study may be requested from the authors.
