# Corrigendum to: Fibulin‐4 as a potential extracellular vesicle marker of fibrosis in patients with cirrhosis https://doi.org/10.1002/2211-5463.13842

**DOI:** 10.1002/2211-5463.13885

**Published:** 2024-08-22

The author affiliations of the paper by Terai *et al*. [1] contains a typographical error:

2. Future Medical Research Center for Exosome and Designer Cells (F‐DEC), Niigata University Japan

The correct affiliation acronym is shown below:

2. Future Medical Research Center for Exosome and Designer Cells (F‐EDC), Niigata University Japan

